# Weighted entropic associative memory and phonetic learning

**DOI:** 10.1038/s41598-022-20798-0

**Published:** 2022-10-06

**Authors:** Luis A. Pineda, Rafael Morales

**Affiliations:** 1 Universidad Nacional Autónoma de México, IIMAS, 04510 Mexico City, Mexico; 2 Universidad de Guadalajara, SUV, 44130 Guadalajara, Mexico

**Keywords:** Psychology, Mathematics and computing

## Abstract

The Entropic Associative Memory (EAM) holds declarative but distributed representations of remembered objects. These are characterized as functions from features to discrete values in an abstract amodal space. Memory objects are registered or remembered through a declarative operation; memory recognition is defined as a logical test and cues of objects not contained in the memory are rejected directly without search; and memory retrieval is a constructive operation. In its original formulation, the content of basic memory units or cells was either on or off, hence all stored objects had the same weight or strength. In the present weighted version (W-EAM) we introduce a basic learning mechanism to the effect that the values of the cells used in the representation of an object are reinforced by the memory register operation. As memory cells are shared by different representations, the corresponding associations are reinforced too. The memory system supports a second form of learning: the distributed representation generalizes and renders a large set of potential or latent units that can used for recognizing novel inputs, which can in turn be used for improving the performance of both the deep neural networks used for modelling perception and action, and of the memory operations. This process can be performed recurrently in open-ended fashion and can be used in long term learning. An experiment in the phonetic domain using the Mexican Spanish DIMEx100 Corpus was carried out. This corpus was collected in a controlled noise-free environment, and was transcribed manually by human trained phoneticians, but consists of a relatively small number of utterances. DIMEx100 was used to produced the initial state of the perceptual and motor modules, and for testing the performance of the memory system at such state. Then the incremental learning cycle was modelled using the Spanish CIEMPIESS Corpus, consisting of a very large number of noisy untagged speech utterances collected from radio and TV. The results support the viability of the Weighted Entropic Associative Memory for modelling cognitive processes, such as phonetic representation and learning, for the construction of applications, such as speech recognition and synthesis, and as a computational model of natural memory.

## Entropic associative memory

The Entropic Associative Memory (EAM)^1^ is a novel declarative but distributed model of associative memory. EAM consists on a set of Associative Memory Registers (AMRs) implemented as finite bi-dimensional arrays or tables. AMRs hold functions in an abstract amodal space standing for the remembered objects. The domain and codomain of such functions are represented by the columns and the rows of the tables, respectively; and the functional assignment is represented by marked cells at the corresponding intersections. Functions representing objects can be overlapped on AMRs and the object held is a relation; as cells in AMRs can contribute to the representation of more than one function, and functions can share memory cells with other functions, the resulting representation is distributed. The maximum number of functions that can be stored in a table of *n* columns and *m* rows is $n^m$.

The system defines three basic operations in relation to a cue: memory register, memory recognition and memory retrieval, which are named $\lambda$, $\eta$ and $\beta$, respectively. Each AMR of size $n \times m$ has an auxiliary register of the same dimension that is used to place the memory cue, and to hold the object retrieved from the memory. In the basic model, the $\lambda$-register operation is defined as the logical inclusive disjunction between the contents of the cells of the auxiliary register (i.e., holding the function to be input) and the current content of the corresponding cells of the AMR. The $\eta$-recognition operation computes whether or not the cue is included in an AMR at the given state, and is defined as the material implication between a cell in the auxiliary register and its corresponding cell in the AMR, for all cells in the tables. Finally, the $\beta$-retrieval operation uses $\eta$-recognition such that if the cue in the auxiliary register is included in the AMR, $\beta$-retrieval selects a value from each of the AMR’s columns randomly, using a probability distribution centered on the cue, and the novel retrieved function is placed on the auxiliary register instead of the cue.

The distributed representation in the AMR holds a large number of objects that can be potentially rendered in relation to a given cue; hence, the memory is indeterminate and has an associated entropy. In the basic model the entropy is defined as the average indeterminacy of the columns –i.e., of selecting one value of an argument among all its possible values– as follows:

$$\begin{aligned} e(r) = -\frac{1}{n} \sum _{i=1}^{n} \log _2(\nu _i) \end{aligned}$$

where *r* is the relation held in the AMR; $\nu _i$ is the inverse of the number of values assigned to the argument $a_i$ in *r*; and *n* is the number of arguments of *r*. If the value of an argument $a_i$ is not defined the function is partial and the corresponding column has no marks on it; in this case $\nu _i = 1$ as the column is nevertheless fully determined. A function is a relation that assigns at most one value to each argument, hence it is a fully determinate object, and its entropy is zero.

The $\lambda$-register operation is productive or generalizes. The number of functions included in an AMR is the number of combinations that can be formed by taking one value of an argument at a time, for all the values of all the arguments. Let $F_T$ be the set of all functions in the AMR at a given state and $|F_T|$ its cardinality. As the entropy is the average indeterminacy of the *n* columns of the AMR $|F_T| = (2^e)^n$ or $|F_T| = 2^{en}$. Let $F_R$ be the set of functions input explicitly through $\lambda$-register, and $F_P$ the productivity of the representation, i.e., the set of functions that are generalized from $F_R$. Clearly, $|F_P|=|F_T|-|F_R|$. Both $|F_T|$ and $|F_P|$ are huge numbers, even for relatively small values of *e* and *n*, and are much larger than $|F_R|$, the units that are actually registered in the memory. The functions in $F_P$ can be thought of as representations of the potential or latent objects stored in the AMR that “emerge” from the distributed representation.

A particular implementation of EAM includes a finite set of Associative Memory Registers. In the basic scenario, each AMR holds the representation of a set of objects of the same class. In previous work we used ten AMRs for holding the representations of the ten digits^1^; and 47 AMRs for holding the representations of capital and lower case letters and digits^2^. We also showed that AMRs can hold representations of more than one class of objects simultaneously, with satisfactory precision and recall, but a moderate increase of the entropy. In particular, we showed that an AMR can hold the representations of two digits at the same time^1^, and that the representations of capital and lower case letters with different shapes can be included in the same AMR, and use 36 AMRs instead 47 in this latter domain^2^.

## Weighted entropic associative memory

In this paper we introduce and extension of the EAM that we call Weighted Entropic Associative Memory (W-EAM). Instead of the inclusive logical disjunction, the $\lambda$-register operation used in W-EAM increases by one the value of all the cells in the AMR corresponding to the cells in the auxiliary register that are used by the cue. In this setting, the content of each column of the AMR can be thought of as a probability distribution in which the probability mass allocated to each cell is its weight divided by the sum of all weights in the column. This distribution is referred to here on as $\Psi$. An AMR is then thought of as a parallel array of distributions $\Psi _i$, for all arguments or columns *i*. The probability mass of individual cells is also used to assess the sensitivity of the memory to accept cues and the strength or intensity that a cue should have to be accepted. These two complementary aspects contribute to fine tune the $\eta$-recognition operation to the kind of data to be stored.

The inclusion of weighted AMRs changes the indeterminacy of the memory. In particular, as different values associated to weighted cells generate differences in the likelihood of selecting cells in memory retrieval, the indeterminacy of the columns, and of the memory as a whole, is reduced. Let $w_{ij}$ be the accumulated weight of value $v_j$ in the argument $a_i$. The accumulated weight of the column *i* is

$$\begin{aligned} W_i = \sum _{j = 1}^m w_{ij} \end{aligned}$$

The probability mass of the cell at row *j* in the column *i* is:

$$\begin{aligned} p_{ij} = {\left\{ \begin{array}{ll} w_{ij}/W_i&{}\text {if } W_i \ne 0\\ 0&{}\text {otherwise} \end{array}\right. } \end{aligned}$$

The entropy of the column is Shannon’s entropy directly, as follows:

$$\begin{aligned} e_i = -\sum _{j=1}^m p_{ij}\log _2(p_{ij})\text{ where } \log _2(p_{ij}) = 0\text{ if }p_{ij} = 0 \end{aligned}$$

The entropy of the whole AMR is the average entropy of all the columns, as in the original formulation:

$$\begin{aligned} e = \frac{1}{n}\sum _{i=1}^n{e_i} \end{aligned}$$

The number of functions stored in an AMR at a given state is the number of combinations that can be formed by taking one value with weight greater than zero for each argument at a time, as in the basic case, but in the W-EAM model there are functions more salients than others due to the uneven weights of the cells in the column, and although $|F_T|$ is still $2^{en}$, the entropy is reduced; hence, the expected $|F_T|$ and $|F_P|$ are lower than in the corresponding basic EAM case.

## Memory operations

The memory operations defined in the originally definition of EAM^1^ are generalized as follows: Let the sets $A = \{a_1,...,a_n\}$ and $V = \{v_1,...,v_m\}$ be the domain and the codomain of a weighted relation $r: A\rightarrow V$ stored in an AMR, and let the function $R: A \times V \rightarrow \{0,..., l\}$, where *l* is an integer greater than zero, specify the weights of *r*, such that $R(a_i, v_j) = w_{ij}$ and $w_{ij} \ne 0$ if and only if $(a_i, v_j)$ is in the relation *r*. Let $r_f$ and $r_a$ be two arbitrary relations from *A* to *V*; $f_a$ a function with the same domain and codomain; and $\Psi _i$ for $1 \le i \le n$ the probability distribution defined by the weights assigned to $(a_i,v_j)$, for all $1 \le j \le m$. The operations are defined as follows:

- Memory Register: $\lambda (r_f,r_a) = q$, such that $Q(a_i,v_j) = R_f(a_i,v_j) \bigoplus R_a(a_i,v_j)$ for all $a_i \in A$ and $v_j \in V$ –i.e., $\lambda (r_f,r_a) = r_f \bigoplus r_a$.
- Memory Recognition: $\eta (r_a,r_f,\iota ,\kappa ,\xi )$ is true if $R_a(a_i,v_j) \rightarrow g(R_f(a_i,v_j))$ for all $v_j$ for at least $n-\xi$ arguments $a_i$ of $r_f$ (i.e., material implication relaxed by $\xi$) and $\rho \ge \kappa \Omega$, and false otherwise, such that:
  - $\omega _i=\frac{1}{k}\sum _{j = 1}^mR_f(a_i,v_j)$ where *k* is the number of cells in column *i* in $r_f$ such that $w_{ij} \ne 0$; i.e., the average weight of the argument $a_i$ among all the none-zero cells in the column;
  - $g(R_f(a_i,v_j))=1$ if $R_f(a_i,v_j) \ge \iota \omega _i$, and 0 otherwise;
  - $\Omega = \frac{1}{n}\sum _{i = 1}^n \omega _i$; the average weight $\omega _i$ of all columns *i*;
  - $\rho =\frac{1}{n}\sum _{i = 1}^nR_f(a_i,v_{cue})$ where $v_{cue}$ is the value $= v_j$ of the argument $a_i$ in the cue $R_a(a_i,v_j)$; i.e., the weight of the cue;
- Memory Retrieval $\beta (f_a,r_f,\sigma ) = f_v$ such that if $\eta (r_a,r_f,\iota ,\kappa ,\xi )$ holds $f_v(a_i)$ is some $v_j$ that is selected randomly from $r_f(a_i)$ –i.e., the column *i*. Let $\zeta$ a normal distribution centered at the cue $f_a(a_i)$ and standard deviation $\sigma$. Let $\Phi _i$ be the scale product of $\Psi _i$ and $\zeta _i$. $f_v(a_i)$ is selected randomly from $\Phi _i$ for all the arguments *i*. If $\eta (r_a,r_f,\iota ,\kappa ,\xi )$ does not hold, $\beta (f_a, r_f,\sigma )$ is undefined –i.e., $f_v(a_i)$ is undefined– for all $a_i$.

In the original presentation of EAM, the operator $\bigoplus$ was defined as the logical inclusive disjunction operation; the parameter $\xi$ was not included formally although it was used in the experiments, and $\eta$-recognition required that all features of a cue were accepted for the cue to be accepted. $\Psi$ was an implicit uniform distribution on all none zero cells in the column; $\zeta$ was a triangular distribution centered on the cue covering the whole column, and neither $\iota$, $\kappa$ and $\sigma$ were included –the first two can be thought of as zero. In W-EAM the $\lambda$-register operation is the arithmetic sum, rendering a distribution $\Psi _i$ on all columns of the AMR. Our experiments show that this distribution is in general not uniform for all non-zero values, as shown below.

We showed in our previous work that recognition failure or the production of false negatives when cues are poor or incomplete may be due to the rejection of a small percentage of the features representing the object. Hence, including the relaxation parameter $\xi$ may help to increase the recall without impacting heavily on the precision. This condition also allows the memory to be resilient to local degradation of the physical substratum or medium, a property that distributed representations should exhibit. Conversely, false positives can be produced easily if there are not restrictions on the strength of the memory cells involved in memory recognition. For this we introduce the parameters $\iota$ and $\kappa$. The first modulates the minimum weight that a cell of the memory register that is hit by the cue must have, for all columns: increasing its value makes the memory more selective favoring precision at the expense of recall, and lowering it produces the reverse effect. If at least one the cell of the memory that is hit by the cue has a lower value than the threshold established by the product $\iota \omega _i$, the argument *i* of the cue is rejected and the whole cue is rejected too –unless it is allowed by the value of $\xi$. Hence, $\iota$ modulates the sensitivity of the memory. Analogously, if the strength of cue, characterized by $\rho$, is lower than the minimum threshold established by $\kappa \Omega$, the cue is rejected too. The expectation is that the combination of the values of the parameters $\iota$, $\kappa$ and $\xi$ allows to fine tune the system for the particular application domain.

The memory recognition operation needs to face that a cue may be accepted by more than one AMR. The resolution of this ambiguity is crucial for the performance of the system. For this we consider that the lower the entropy of an accepting AMR, the more determined the distributed representation held in it, and the more likely to be the right one to choose in a memory recognition operation. We also take into account that the higher the weight of the cue –i.e., $\rho$– the most likely that such AMR should be chosen. For the selection of the most likely responding AMR these two parameters are pondered and the AMR selected is the one for which the product of its entropy and $1/\rho$ is the lowest. This is an instance of a Bayesian decision where $1/\rho$ corresponds to a likelihood and the entropy of the AMR to a prior.

In the basic EAM the value of the cue at each column is the reference for selecting the value of the corresponding attribute of the recovered object. In the present W-EAM model, representing the information in the column as a random distribution requires that the cue is represented in a compatible form. For this, the cue is thought of now as a normal distribution $\zeta$ on each column, that is centered on the actual value of the cue at the column, ranging over some standard deviation $\sigma$, measured in cells above an below the cue. The modulation of the distribution $\Psi$ by the distribution $\zeta$ renders the combined distribution $\Phi$ in which the cells close to the cue are favored. The present definition of the $\beta$-retrieval operation obeys also the Bayesian intuition that a decision should ponder the evidence, in this case the component of the cue, represented by the distribution $\zeta$, with the prior, represented by the distribution $\Psi$.

## Related work

EAM and W-EAM differ from associative memory models developed within sub-symbolic systems, such as the Artificial Neural Networks (ANNs), in the tradition of Hopfield’s model^3^ and related work^4–14^, although there are some early relevant exceptions^15^. In our previous work we have discussed extensively such differences in several dimensions^2^. A comparison of our approach with ANNs models in Hopfield’s paradigm that use an energy function is summarized in Table 1.

**Table 1:** Summary of differences between EAM and W-EAM and associative memory models developed within the Artificial Neural Networks paradigm.

Property	EAM and W-EAM	ANNs and related models
Representational format	Declarative but distributed such that the relation between cells in AMRs and memory contents is *many-to-many*	Sub-symbolic, embedded in numerical matrices
Memory operations	Declarative manipulations on cells and columns of AMRs	Matrix additions and multiplications operations
Productivity of representation	Emerging objects of the set $F_P$	The memory is oriented to store and match patterns and there is no productivity
Memory register	Produces the abstraction of the input cue with the content of the memory	Updates a numerical matrix of weights
Memory recognition	Test the inclusion of the cue in the memory through the logical material implication	Performs a numerical search until the cue and the product of a matrix operation converges
Memory retrieval	Constructive operation that produces novel objects on the basis of a complete or a partial cue and the memory content	Reproductive or photographic operation that reproduces a previously stored object on the basis of a complete or a partial cue
Demarcation between auto and hetero-associativity	Weak	Strong
Rejection of cues not contained in the memory	Direct without search	Rejection by failing to find, implementing a form of the closed-world assumption
Parallelism	Direct parallel manipulations of cells and columns	Parallel computation of matrix operations
Main functional parameter	Entropy –no energy function is used	Energy function –the entropy has no functional role
Memory capacity and use	A function of the entropy	Depends of the number of local minima of the energy function
Demand of memory and processing resources	Low	High

The W-EAM model is used in the present investigation for storing phonetic information, for supporting phonetic recognition and synthesis, and for phonetic learning. Phones are commonly modelled with sub-symbolic structures; for instance through Hidden Markov Models, as in the Sphinx framework^16^, and more recently, by means of deep neural networks, as in the Kaldi project^17^. Associative memories, such as Willshaw’s^18^ and Pam’s^19^, have been used for word and sentence recognition^20^, and for the integration of language, vision and action^21^, but the phonetic recognition module of such systems used Hidden Markov Models too. Our system uses two levels of phonetic processing: a sub-symbolic non-declarative level, implemented with a basic deep-recurrent neural networks model^22^, and a symbolic declarative level using the AMRs and its associated memory operations. The incorporation of an associative memory into the phonetic processing architecture suggests the use of the phonetic units stored in the memory to recognize novel speech, and also to synthesize novel units that can be used to improve the performance of the analysis and synthesis modules, and of the memory operations. In this paper we introduce a learning model to such an effect.

## Architecture of the memory system

The memory system includes three representational levels from bottom to top, as follows:

1. The modal specific input and output representations of the domain. In the present application to phonetics the objects at this level are MFCCs commonly used in speech processing; these consist of vectors of real numbers –26 in our experiments– standing for speech segments. Each MFCC vector represents $25\,\text{ ms }$ of speech, the signal is sampled every $10\,\text{ ms }$ –so there is a $15\,\text{ ms }$ overlap between contiguous samples– and phones are represented as sequences of 8 MFCCs lasting $95\,\text{ ms }$, hence each unit has $p=208$ features or characteristics with real values. The average length and standard deviation of Mexican Spanish phones in the DIMEx100 Corpus^23^ are $69.9\,\text{ ms }$ and $28.9\,\text{ ms }$, respectively; shorter phones are adjusted to $105\,\text{ ms }$ by filling in the previous and following speech segments in relation to their centers, and two samples are generated; for larger phones more samples are produced.
2. The abstract amodal representations of the individual modal-specific units. These are functions or attribute-value structures standing for their corresponding representations in the first level. In the present study the cardinality of the domain of these functions is $n=32$. This value was determined through preliminary experiments, and constitutes a good compromise between the characteristics of the AMRs and the configurations of the analysis and synthesis modules used for registering and retrieving the remembered objects, as discussed below.
3. The abstract amodal distributed representations held in the AMRs. These stand in a *many-to-many* relation with the representations in the second level. The AMRs have *n* columns and *m* rows, to store and retrieve the functions at the second level. The value of each attribute at this level is the quantized value, in *m* levels, of the corresponding argument in the intermediate representation. The parameter *m* is determined empirically, as explained below.

The system’s architecture includes:

- The input and output buffers where the first level modality specific representations are placed.
- The analysis module that maps the representations in the first into the second level, and the synthesis module that makes the reverse mapping.
- The AMRs registers holding the distributed representations at the third level. In the present study there is an AMR for each kind of phone,

The $\lambda$-register operation inputs the cue to its corresponding AMR in a supervise manner through the analysis module and its associated auxiliary register. The $\eta$-recognition operation presents the cue to all AMRs simultaneously, and the results of the tests are codified in their corresponding auxiliary registers. The $\beta$-retrieval operation uses $\eta$-recognition, and places the recovered objects in the auxiliary registers of the responding AMRs.

**Figure 1:**
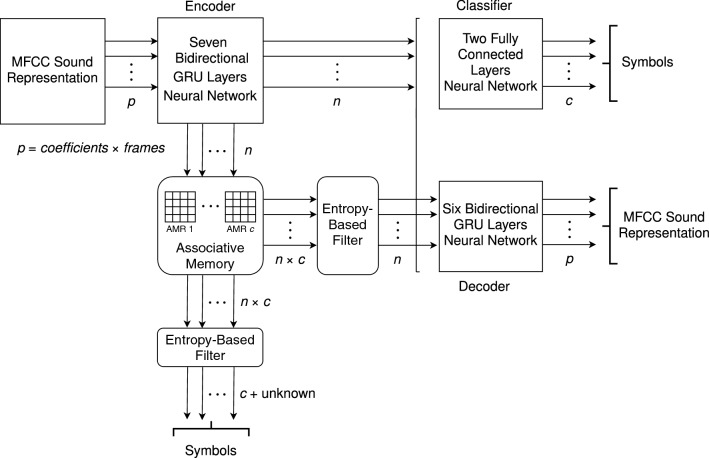
System architecture.

The functional architecture, including the three representational levels, is illustrated in Fig. 1. The analysis module is implemented with an *encoder*^24^, with a seven layers Bidirectional Gated Recurrent Unit (GRU) neural network^25^. The outputs of the encoder have real values which are quantized and normalized in relation to the number of rows of the AMRs. The objects recovered by the $\beta$-retrieval operation are the inputs to the *decoder*. This module is defined as a six layers bidirectional GRU neural network, that maps the *n* arguments of the intermediate representation into the *p* features of the output MFCC. The encoder and the decoder constitute an *autoencoder*. There are *c* AMRs, one for each class of phone. There is also a classifier, which assigns the functions in the intermediate representation to one among the *c* phones; it is implemented as a two fully connected layers neural network mapping the *n* arguments into the *c* classes, and it is used to train the encoder, whereas the outputs of the trained encoder are used to train the decoder. The seven layers that compose the encoder start with an initial size of $1024\times 8$, which is divided by half until they reach a layer of $32\times 8$; then the sequence is discarded, yielding a layer of size 32. The layers in the decoder follow a similar pattern, but starting with a Repeat layer of $32\times 8$ followed by a $1024\times 8$ bidirectional GRU, and ending with an output layer of $26\times 8$.

## Phonetic memory for Mexican Spanish

Next we present the application of the W-EAM to phonetic representation and learning. The initial state of the AMRs was produced using the Corpus DIMEx100 for Mexican Spanish^23^. The corpus is constituted by 6000 utterances, recorded by 100 speakers, in a noise-free controlled environment, using a high quality microphone. Each speaker recorded 60 utterances, 50 assigned individually and 10 recorded by all the 100 speakers –the common utterances. The corpus reflects the representation of phones in the language and is phonetically unbalanced. It was tagged manually by trained phoneticians using the Mexbet alphabet in its basic transcription level, which defines 22 phones^23^, as shown in Tables 2 and 3.

**Table 2:**
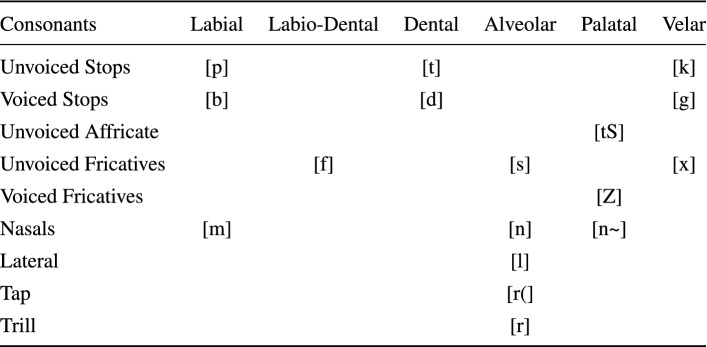
Consonants of the Mexbet-22 phonetic alphabet for Mexican Spanish.

**Table 3:**
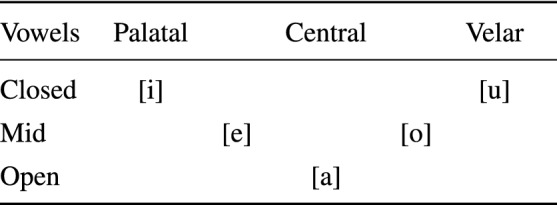
Vowels of the Mexbet-22 phonetic alphabet for Mexican Spanish.

The memory system was configured using 4000 individual utterances. The remaining 1000 individual and the 1000 common utterances were used to test the overall phonetic recognition performance of the system, as described in the “Phonetic recognition” section. The 4000 utterances were divided in three mutually exclusive partitions, as follows:

- Training Corpus (*TrainCorpus*): For training the analysis and synthesis modules (70%).
- Remembered Corpus (*RemCorpus*): For filling up the Associative Memory Registers (20%).
- Test Corpus (*TestCorpus*): For testing the classifier (encoder-classifier), the autoencoder (encoder-decoder), and the memory register (10%).

The memory system was set up using the following procedure:

1. Train the classifier and the autoencoder using the *TrainCorpus*. This was divided into a $80\%$ training and a $20\%$ validation partitions, and the autoencoder and classifier were tested with the totality of the *TestCorpus*.
2. Determine the optimal size *m* of rows of the 22 individual AMRs, filling the AMRs with the totality of the *RemCorpus*. Determine the performance of the system as a whole, for the AMRs of size $32 \times m$ where $m=2^r$ for $0\le r\le 10$. Select the size of optimal AMR, i.e., $32 \times m$, with the best performance in the domain.
3. Determine the performance of the optimal size AMR with different amounts of *RemCorpus*: $1\%$, $2\%$, $4\%$, $8\%$, $16\%$, $32\%$, $64\%$ and $100\%$, both for the average of the individual AMRs and for the system as a whole.
4. Use the register with the optimal size—selected in (2)—with its optimal filling level—determined in (3)—for phonetic recognition and learning, as described below.
5. Test the procedure with a standard 10-fold cross-validation procedure.

The behavior of the system depends greatly on the choice of the recognition parameters’ values. The increase of $\iota$ and $\kappa$ impact on the “force” that the cues must have to be accepted, reducing the number of false positives, but at the expense of rejecting true positives, with the subsequent increase of false negatives, and the reduction of the recall. The parameter $\xi$ works in the opposite direction, increasing the recall but at the expense of the precision; and the parameter $\sigma$ impacts on the similarity of the cue and the object constructed in a memory retrieval operation: the lower its value, the larger their similarity, and when $\sigma =0$ the object retrieved is a “photographic copy” of the cue. To appreciate the impact of the parameters six scenarios were studied, as shown in Table 4.

**Table 4:** Parameters values of the six scenarios.

Scenario	$\iota$	$\kappa$	$\xi$	$\sigma$
I	0	0	0	0.5
II	0.3	0	0	0.5
III	0	1.5	0	0.5
IV	0	0	0	0.1
V	0.3	1.5	0	0.1
VI	0.3	1.5	1	0.1

**Figure 2:**
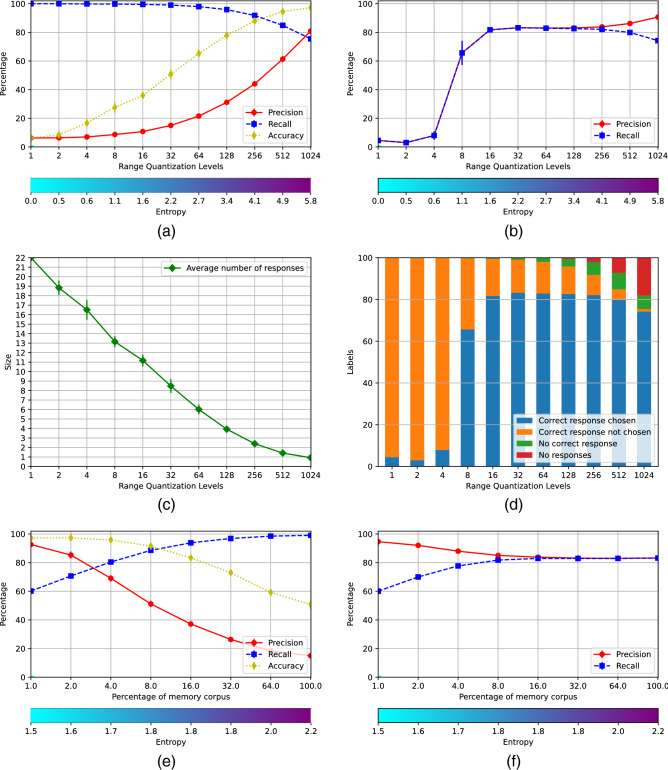
Memory scenario I.

Figure 2 shows the results of the basic scenario, generated using the parameters $\iota = 0$, $\kappa = 0$, $\xi = 0$ and $\sigma = 0.5$. The accuracy of the classifier is 0.821 and the root mean squared error of the autoencoder is 8.93. Figure 2a shows the average performance of the 22 individual AMRs at the different values of *m*. The precision, the recall and the accuracy are shown with the red, blue and yellow lines, respectively. These parameters are defined in the standard form^26^, although taken into account that if all cues are rejected, all true positives are missed out and the recall is 0, but there are neither false positives and the precision is 1.

The entropy at the different number of rows is shown by the bar at the bottom. The recall is very high for values of *m* up to 64 but the precision is very low. In this range most AMRs accept a large number of cues, as can be seen in Fig. 2c, producing a large number of false positives, with the consequent decrease of the precision. For values of *m* larger than 64 the number of responding AMRs lowers, the precision rises and the recall decreases, and there is good compromise between precision and recall in AMRs with 1024 rows. The accuracy graph shows that individual memories with *m* larger than 256 are very good at rejecting cues that belong to other categories, 21 out of 22 cues in *TestCorpus*, for each memory.

The precision and the recall of the final decision made by the system is illustrated in Fig. 2b. There are no true negatives at the system level and accuracy coincides with recall. The system’s choice is made pondering the strength of the cue $\rho$ with the entropy of the AMRs that accepted it, assuming that the strongest the cue and the lowest the entropy of the AMR, the more likely that such AMR is the right one. The precision and the recall are both very low for small values of *m*; then there is sudden jump at $m=8$, and a further increase for $m=16$, where both the precision and the recall reach a level above of 0.8; this level is sustained until $m=64$ when the precision and the recall start to diverge. The decision is illustrated further in Fig. 2d. The blue bars indicate that the right AMR did respond and was selected by the decision procedure; the orange bars show that the right AMR responded but it was not chosen. The green bars show that the right AMR did not respond producing a false negative; and the red ones indicate that the cue was rejected by all AMRs, given rise to a false negative too. For instance, the choice when $m=16$ involved selecting one among 11 responding AMR; nevertheless, the choice was the right one about $80\%$ of the times. Figure 2b and d indicate that the best AMR’s size for this domain is $m=32$. Hence, an operational memory system should include registers of size $32\times 32$.

The next question to assess is the amount of *RemCorpus* that must be feed in into the AMRs for optimal recognition. This is shown in Fig. 2e and f, for individual AMRs and for the system as a whole, respectively. Figure 2e shows that individual memories perform best with a very small percentage of the filling corpus, and that the best trade-off between precision and recall occurs when the AMRs are filled with around $3\%$, whereas for larger amounts such compromise decreases significantly. However, the performance of the system as a whole, shown in Fig. 2f, reaches a little above 0.8 for both the precision and the recall with $8\%$ of *RemCorpus*, and remain at that level then on. Hence, for actual phonetic recognition, AMRs of size $32\times 32$ filled up with at least $8\%$ of the remembered corpus should be used, although the graph shows that an unbounded amount of new information can be registered in the AMRs without harming the system performance.

**Figure 3:**
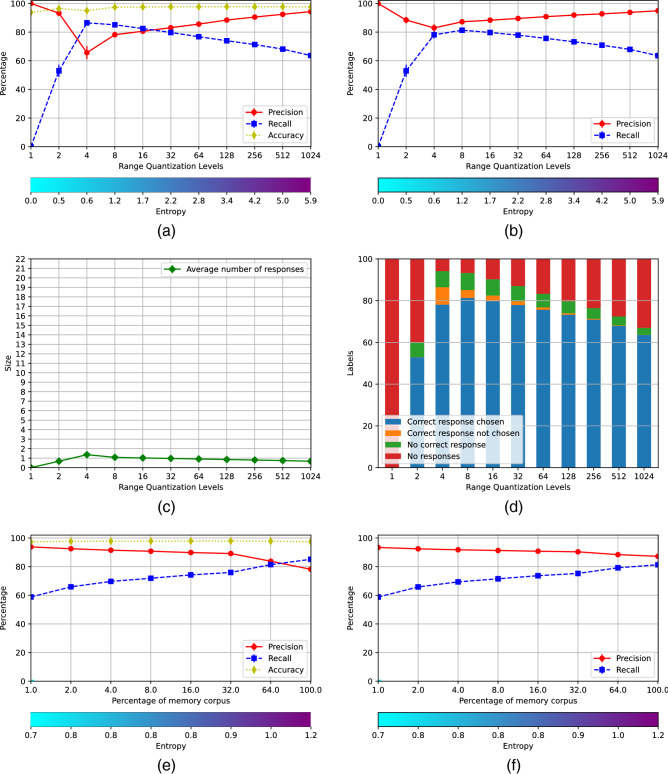
Memory scenario V.

Figure 3 shows the scenario generated using the parameters $\iota = 0.3$, $\kappa = 1.5$. $\xi = 0$ and $\sigma = 0.1$. The accuracy of the classifier is 0.817 and the root mean squared error of the autoencoder is 8.94. The performance of the individual memories is shown in Fig. 3. The the precision and the recall for $m=1$ are 1 and 0, respectively. However, the recall is increased up to almost 0.9 when the registers have only 4 rows, although with a slight decrease of the precision, which grows steadely for larger values of *m*, although the recall decreases slightly all the way too. In this scenario the AMRs respond much more selectively, as shown by the their very high accuracy, and only one responds to most cues for values of $m\ge 4$, as can be seen in Fig. 3c. Nevertheless Fig. 3b and d show that the final decision made by the system is very good even for registers with $m=4$. The best compromise between precision and recall is achieved with $m=8$, in contrast to scenario I where the optimal number of rows is 32. Figure 3e and f show the performance of registers of size $32\times 8$ with different amounts of *RemCorpus*. The precision, recall and accuracy of the individual registers improve considerably in relation to scenario I, with a significant reduction of the required AMR’s size, and the performance of the system as a whole reaches a precision of almost 0.9 with recall of 0.81 when the full of the remembered corpus is used. This trend could be sustained if novel units of the class are registered. These numbers are within the state of the art for phonetic recognition in Mexican Spanish, and are pretty good for the overall technology^27^. The parameters values also impact in the entropy, which lowers from 2.2 in the best recognition in scenario I in Fig. 2f to 1.2 in scenario V in Fig. 3. The choice of parameter’s values transferred the load of decision making from a system’s mechanism, that had to decide between a number of responding AMRs in scenario I, to $\eta$-recognition, that becomes much more selective, in scenario V.

## Phonetic recognition

**Figure 4:**
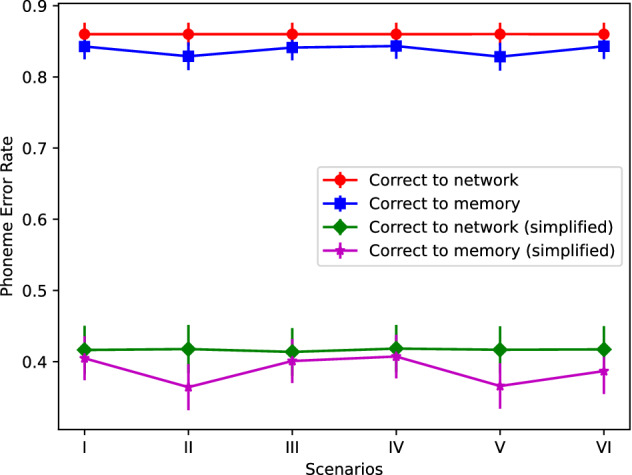
Phoneme Error Rate between the strings recognized by the AMRs and the string recognized by the classifier in relation to the correct string, respectively, for the six scenarios.

The memory system was assessed through a speech recognition task. In particular, we analyzed whether there is a significant recognition performance increase using W-EAM in relation to recognizing the input speech with the neural networks only, all other aspects of the task being equal. For this, we compared the distances between the strings produced by the memory and the classifier, *Mem2Utt* and *Net2Utt*, respectively, to the phonetic transcription of the utterance in DIMEx100 using the Phoneme Error Rate (FER), defined as:

$$\begin{aligned} FER = \frac{I+D+S}{I+D+S+L} \end{aligned}$$

where *I*, *D*, and *S* are the insertions, deletions, and substitutions needed to transform the produced string of phones into the correct one, and *L* is the length of the correct string. This distance is 0 if the compared strings are the same, and approaches to 1 for strings that are very different different from the right one. The 2000 utterances of DIMEx100 set apart from the *TrainCorpus*, *RemCorpus* and *TestCorpus* were used for this test. The scenario V produces the best recognition performance, which is slightly better than scenario II, as shown in Fig. 4. Due to the sampling rate, an average of 8 predictions per actual phone is expected in the string produced by the classifier, that accepts everything and, as seven out of eight instances have to be modified, the expected distance is

$$\begin{aligned} FER = \frac{7L}{7L+L} = 7/8 = 0.875 \end{aligned}$$

On the other hand, the memories can reject cues producing shorter strings.

Figure 4 also shows the *FER* of strings produced by the memory and the networks but simplified using the conditional probability distributions between phones generated from the collection of bigrams extracted from DIMEx100. We calculated the conditional probability for a phone *b* be correct given the previous phone *a* (already accepted) and the next phone *c* as

$$\begin{aligned} p(b|a,c) = wp(b|a) + (1-w)\frac{p(c|b)p(b)}{p(c)}, \end{aligned}$$

where *p*(*b*) and *p*(*c*) are calculated from the frequencies of *b* and *c* in DIMEx100, and $w=0.5$. Phone *b* was accepted if $p(b|a,c) > p(b)$. The simplified distances *SMem2Utt* and *SNet2Utt* preserve the same relation, showing the gain of using the W-EAM versus computing the phones using the networks alone.

Table 5 shows an instance of a recognized utterance: its orthographic and phonetic transcriptions in DIMEx100; the strings produced by the memory and the network, with the corresponding Levenshtein’s distances to the correct string, and also the distance between the output of the network to the output of the memory *Net2Mem*, both in their original and simplified forms. As can be seen, *Mem2Utt* is significantly lower than *Net2Utt*, and *Net2Mem* shows the improvement of the string produced by the memory in relation to the one produced by the network. The gain is mainly due to the direct rejection by the memory of segments in the input speech, and to the fact that the encoder always approximates the input segment to the closest phone, producing a large number of false positives. Table 5 shows only the phonetics processing level of the full speech recognition task, and although is difficult to assess the load that is carried on by each module of a full speech recognition system, the amount of work performed by the pronunciation and the language model processing levels is large^23^. We leave the construction of a full speech recognition system for further work.

**Table 5:** Example of a recognized utterance and the distances between the strings recognized by the memory and the network to the original phonetic transcription.

Concept	String	Parameters
Utterance’s orthographc transcription	Este es el resultado de ese trabajo	
Phonetic transcription	esteselresultadodesetr(abaxo	Length: 27
Network output	fpndddgieegeeeeeigggsssssttttdeeeeedsssddggeeeeer(r(llrrrrrrrraaaaaess ssssffuuuubblllllllldpptttttr(r(aaaaaaaaaaaaooooooooodddddddddddeee eeeeeeeennngsssseeeeedppppppttr(r(r(r(aaaaaaabbboaaaaaaaaaxxxxxxx xxxxxxxxxxxooooooooafdsdddfpppp	Length: 225 Net2Utt: 199
Memory output	dddieeeeeeiigsssstttdeeeeesssdieeeer(r(llr(rrrrrrraaaar(ssssuuuubbllllllllp tttttr(r(aaaaaaaaaaaaoooooooooddddddddddeeeeeeeeeeessseeeeppppttr( r(r(r(aaaaaaabbbaaaaaaaaxxxxxxxxxxxxxxxoooooopp	Length: 178 Mem2Utt: 153 Net2Mem:51
Simplified network output	ndiegigstdeser(aesfubltr(r(addeensptr(r(abaxad	Length: 41 SNet2Utt: 20
Simplified memory output	digstdeesdier(ar(ubltr(r(addeesetr(r(abaxop	Length: 37 SMem2Utt: 18 SNet2SMem: 14

## Phonetic learning

The memory system can be used for phonetic recognition and synthesis directly, but also to improve its own performance, by enriching the phonetic corpus with the empirical data recognized by the system itself. In this section we present an experiment to such an effect. The learning process was modelled in five incremental stages over the basic state –the *stage 0*– using CIEMPIESS^28^, a large and noisy Mexican Speech corpus collected from radio and TV. Each learning stage has an active phase in which novel phonetic units are recognized and collected using the current state of the analysis and synthesis modules, and the current remembered corpus; and a passive phase in which the analysis and synthesis modules are retrained using the new units, and the remembered corpus included in AMRs is enriched with new empirical data, rendering a new state of the system. The process is performed recurrently until the five stages are completed.

The DIMEx100 is significantly unbalanced, and there are very few instances of the less represented units, affecting the performance of the autoencoder and the classifier. For this reason, the classes with a number of instances above the median of the frequencies were cut off, producing a more balanced initial dataset. Then, the learning process was targeted to collect units of the less represented phones, up to $10\%$ above the previous median. Highly represented units were collected too in order to include phonetic units of both the DIMEx100 and CIEMPIES, but limiting the increased up to one tenth of the units of the most represented phone.

The novel speech utterances are sampled from left to right every $10\,\text{ ms }$. All samples are submitted for recognition through the encoder and, whenever they are accepted, are used as cues to the $\beta$-operation. The cue and its corresponding retrieved unit are included in the enriched Corpus used in the next training stage if both belong to the same class. Conversely, windows that are not recognized by the memory system are rejected directly. The set of novel units produced at the active phase of the stage *s* is partitioned in the subsets $Train_s$, $Rem_s$ and $Test_s$. These sets are used in the passive machine-learning phase of stage *s* to generate and test the phonetic machinery at the stage $s+1$. The overall expectation is that the novel units produced at each learning stage improve the performance of the analysis and synthesis machines, and of the memory system as a whole.

**Figure 5:**
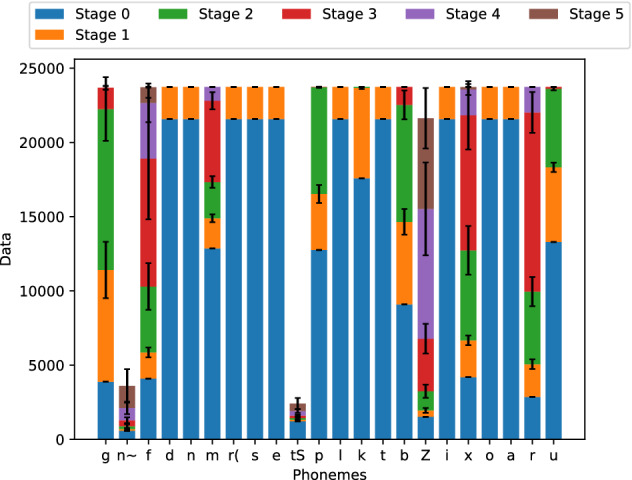
Corpora enrichment for the six scenarios for $\iota$, $\kappa$, $\xi$ and $\sigma$.

The corpus enrichment along the five learning stages is shown in Fig. 5. The lengths of the bars show the average number of phonetic units of all six scenarios at each learning stage for the corresponding phone, and the black lines at the middle stand for the standard deviation. As expected, the instances of the less represented phones are increased significantly in most scenarios, rendering a better balance between classes through the learning process; although these instances are the most sensitive to the parameters’ values, as indicated by the larger standard deviation.

**Figure 6:**
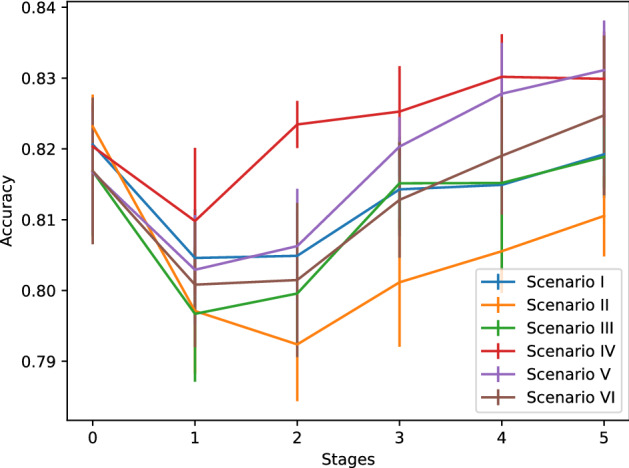
Performance of the classifiers along the five learning stages for the six scenarios.

**Figure 7:**
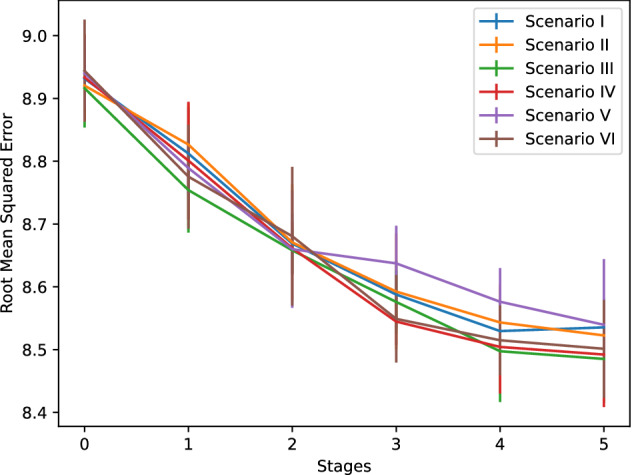
Performance of the autoencoders along the five learning stages for the six scenarios.

The enriched and more balanced corpora impacts on the performance of the classifiers and the autoencoders along the five learning stages, for each of the six scenarios, as shown in Figs. 6 and 7, respectively. The classification performance showed a small decrement in the first and the second stages, due most probably to the differences between the DIMEx100 and CIEMPIESS, but then recovers, and has a significant improvement in the last three stages, reaching up to 0.83 for scenario V and above 0.8 for scenario II, which was the lowest. The six scenarios present a similar decrease of the autoencoder’s root mean square error, with a very small difference between the best and the worst scenarios, which were III and V, respectively. The results suggest a small trade-off between the classifier’s and the autoencoder’s performance. The classification error improves over the results reported for Spanish^27^, both using a small training corpus consisting on 1000 utterances, and a large corpus including 20000 utterances, which are $35.9\%$ and $18.5\%$, respectively. We are not aware of better figures reported for Spanish.

**Figure 8:**
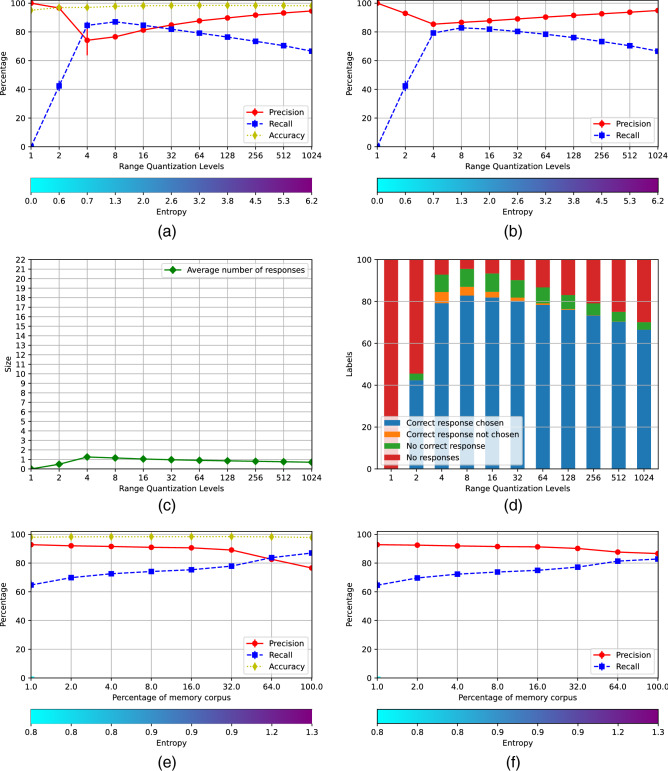
Scenario at the sixth stage of the learning process built upon scenario V.

**Figure 9:**
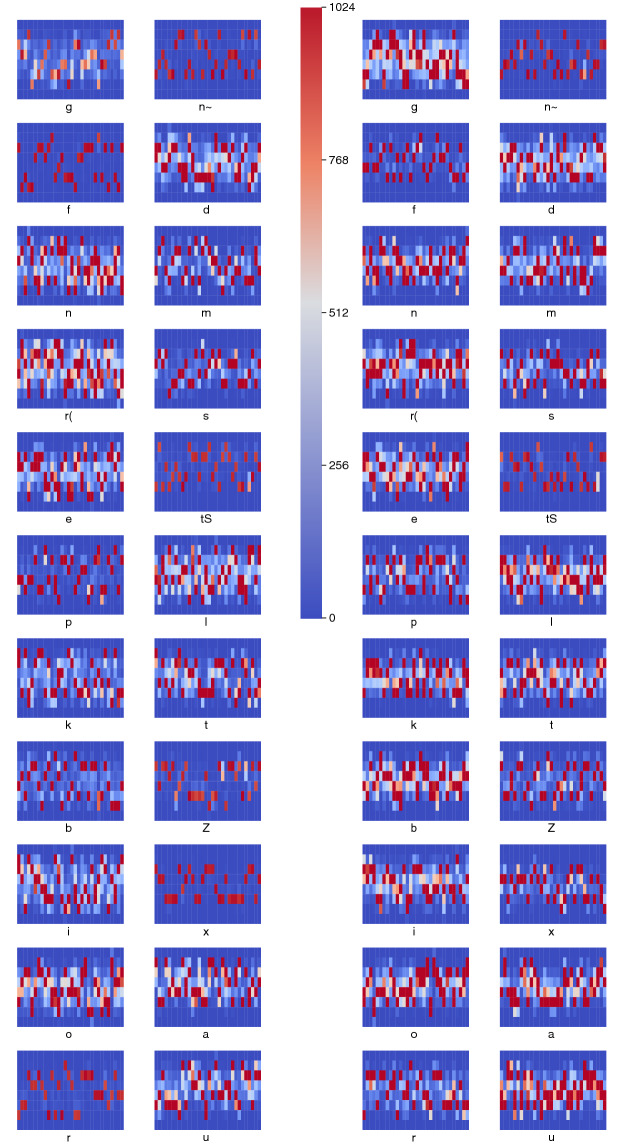
Phonetic memories at the initial (left) and final states (right) of the learning process for the scenario V.

The fifth learning stage of the scenario V is shown in Fig. 8. The learnt information is reflected in the overall improvement the recall of $1.5\%$ with a precision decrease of only $-0.57\%$ of the system as a whole. The improvement can be seen in the size of the blue bars in Fig. 8d, which represent the phones that were accepted by their right AMR, and chosen among other hypothesis by the decision making mechanism; by the decrease of the green bars and yellow bars, which stand for false negatives and false positives, respectively; and also by the decrease of the red bars, which represent phone rejected the all AMRs, and constitute false negatives for the system as a whole. The learning process is also reflected in the phonetic memories held in AMRs. Figure 9 shows the content of the AMRs for the 22 phones in the scenario V, both in its first and last stages, as shown in Figs. 3 and 8, respectively. The dark blue indicates low values of the corresponding cells, and the more intense the red the higher the value of such cells. Both the initial and the last stages have similar overall patterns, but the last stage shows a richest and wider range of shades, which may impact on the recognition finesses.

## Discussion

The W-EAM memory extends the EAM model with a learning mechanism to the effect that cues registered into the AMRs strengthen the memory cells that they use, one for each column. In the new setting, cells frequently used become more active in memory recognition and retrieval. The introduction of weights motivated making explicit the parameters $\xi$ and $\sigma$, which were implicit in the basic model, and the introduction of the parameters $\iota$ and $\kappa$. The former models the sensitivity of the memory cells, which depends exclusively on how often they are used by the $\lambda$-register operation, and constitutes a priori information. Cells with very low sensitivity behave as blanks in the inclusion test defined through the logical material implication of $\eta$-recognition, allowing the system to reject cues that that have attributes with a very low probability mass relatively to the overall distribution in their corresponding columns. Conversely, the $\kappa$ parameter allows to set up the minimum weight or “force” that a cue needs to have to be accepted by the memory. The combination of these parameters, in conjunction with the relaxation parameter $\xi$, allows to fine tune the strength of the cues that are accepted, and to prevent the production of false positives with little force, although to the expense of rejecting true positives units that are weak in relation to the more representative ones. The parameter $\sigma$ allows to control the level of randomness of the $\beta$-retrieval operations, from the photographic case to a wide range of constructions. The parameters values also impact significantly on the optimal size of the AMRs, which may be very small.

The number of function or objects stored effectively in the AMRs at any given state is $|F_T|=2^{en}$, where $F_T$ is the union of the set $F_R$ of functions registered explicitly through the $\lambda$-register operation and the set $F_P$ of functions that emerge from the distributed representation, i.e., $F_T = F_R \cup F_P$. $|F_T|$ and $|F_P|$ are huge numbers, even for relative small AMRs with low entropy. Lowering the entropy reduces the size of operational AMRs and allows to storing a very large number of objects with very economical computing resources and satisfactory performance. These improvements allow to tackle natural domains, such as natural speech, in addition to domains involving conventional symbols, such as digits and letters, as was done with the basic EAM model. The present experiments were performed with standard CPUs using Graphical Processing Units (GPUs), but the memory operations can be performed as massive parallel computations if appropriate hardware that takes advantage of the cell to cell and columns operations is made available.

The functionality and performance of the W-EAM was tested with a case study on phonetic representation and learning. In this context we introduce the notion of phonetic memory for the declarative representation of phones. The experiments show that there is a positive impact on the phonetic recognition performance using the W-EAM, which is assessed by comparing the phoneme error rate between the correct phonetic string and the strings produced by the neural network and by the memory, as shown in Fig. 4. The results show that a phonetic memory fine tunes the hypothesis produced by the network through the rejection of many speech segments, such as those not aligned closed enough to phones; and also of cues that despise of being accepted are too weak. In the present experiments we used the same values for the parameters $\iota$, $\kappa$, $\xi$ and $\sigma$ for all 22 AMRs; a question for further research is whether the overall performance can be improved if optimal values for each phone are used instead.

The phonetic memory allows to collect the phonetic experience, and use such knowledge in service of phonetic learning. This was shown in the experiments in the “Phonetic learning” section in which novel speech input was recognized directly from the input speech, and the individual units were phonetically tagged and time aligned dynamically. Hence, the novel units could be collected and added to the corpus used in the next learning stage, which in turn was used for re-training the neural networks, improving their performance significantly. The enriched corpus included the units recognized and used as cues to the AMRs, as well as the corresponding units that were produced by the $\beta$-retrieval operation and synthesised through the decoder, modelling a negative feed-back phonetic learning loop. The process was developed in five learning stages, but it could be carried on in the long term. The performance of the basic model built with DIMEx100 is within the state of the art for phonetic recognition and the output of the fifth learning stage in Scenario V showed a small improvement over the best figures that we are aware of^27^.

In natural speech recognition, phonetic learning is performed directly from the raw speech signal. The present machinery performs such task and can prevent the need of massive amounts of corpus tagged with statistical tools for phonetic training, such as force alignment, that requires the orthographic transcription of the speech, which is not available in the natural setting. Our results also show that recognition performance and the trade-off between precision and recall in the optimal scenario is sustained regardless the amount of phonetic units included in the AMRs; hence, novel recognized units can be registered directly into the corresponding AMRs, implementing a form of unsupervised learning, in which such units are not used for training but are just remembered. We leave this functionality for further research too. A problem for further study is that the recognition performance of the 2000 DIMEx100 corpus utterances that were used for testing did not improve across the training stages; this may be due to the fact that the stage 0 is the best model for such utterances, and although the stage 5 is a better model for the language as a whole, is less specific to DIMEx100 utterances.

The purpose of the case study in phonetic representation and learning is to show the functionality and potential utility of the W-EAM model with a practical example. For this we use the basic deep neural networks model to implement low level analysis and synthesis modules. We leave for further research addressing the potential interaction of W-EAM with the current *end-to-end* speech recognition trend, including algorithms such as the Connectionist Temporal Classification (CTC)^29^ and the so-called “Listen, Attend and Spell” (LAS)^30^.

A more general issue concerning our overall research program is whether the cognitive architecture of computational agents should include an associative declarative memory in addition to the perception and action systems, which are commonly construed as sub-symbolic. Our model supports the view that both levels are required and contribute to improve the overall cognitive performance, and that memory retrieval is a constructive operation, such that remembered objects are genuine novel constructions, in opposition to the view that the whole of cognition reduces to a neural networks procedural level. This dilemma reflects the opposition between innate versus empirical knowledge, and also between constructivism versus associationism, e.g., Piaget and Bartlett’s versus classical behaviorism. The present model offers a perspective on these conflicting views. The set of remembered objects $F_R$ consists on empirical data that gives rise to the set $F_P$ of potential or latent objects, which emerge as collateral effects of the distributed representation. The objects in $F_P$ are neither innate nor empirical knowledge but provide functional pivots for recognizing novel empirical information, that could not be recognized otherwise, and also for synthesizing novel constructions through the memory retrieval operation. As $F_P$ is usually much larger than $F_R$ there is a large body of potential knowledge that may become actual, even on the basis of a very moderate amount of empirical experience. Recognizing and retrieving information requires that there are objects stored in the memory –nothing can be recognized or retrieved from an empty container. The *tabula rasa* and the *innate knowledge* scenarios correspond to whether the information to bootstrapping the system, which we refer to as $F_{R_0}$, is input through the memory register operation, or whether there is an initial amount of information provided within the genetic constitution of the agent. The former view involves that $F_{R_0}$ is learned in a supervise manner through a very long learning phase, while the latter assumes that there is a platform for launching an unsupervised learning cycle to start with, and favors a faster learning rate. In any case, $F_{R_0}$ gives rise to an initial pivot set $F_{P_0}$ from which novel empirical recollections can be stored, recognized and retrieved. The cognitive architecture should include a set of AMRs, possibly supplied with a small amount of innate knowledge, and a basic set of perception and action networks, genetically given, that enable the system’s bootstrapping; and the performance of the perception, the action and the memory modules, should improve on the basis of empirical information through long term learning. In the present experiment the initial state of the system is given by the DIMEx100 corpus –which was collected in a noise free environment– and the knowledge acquired from CIEMPIESS constitutes empirical knowledge. Training the perceptions and action networks is a paradigmatic associative learning task, but retrieving novel objects from the memory is a constructive process.

From the perspective of the neurosciences, the dual-stream model of the functional anatomy of language^31^ holds that, according to current evidence, there is a spectrotemporal analysis module (at the dorsal Superior Temporal Gyrus) that connects directly with the phonological representation and processing module (at the Mid-post Superior Temporal Sulcus). Then the system diverges into two streams: (1) the Dorsal-stream which maps the phonological information into the sensorimotor interface (at the Parietal-Temporal boundary), where the input from other sensory modalities is collected too, which in turn connects with the articulatory network involving several cortical regions; and (2) the Ventral-stream through which the phonological information is mapped into the lexical interface (at the posterior Middle Temporal Gyrus and the posterior Inferior Temporal Gyrus) and then to the conceptual network, which is widely distributed. The architecture of the EAM model suggests an analogy with the circuit formed by the spectrotemporal analysis module, the phonological module, and the modules and connections in the Dorsal-Stream, in which the phonological module would include a declarative phonetic memory in contrast to current ANNs assumed in neuroscience models. The present model also suggests that there is declarative format in which a very large amount of recollections is stored in an abstract amodal but widely distributed representation.

The so-called sensorimotor integration hypothesis in speech processing sustains that the auditory system impacts critically in speech production and vice versa^32^. The suggestion is that the predictions made by the motor system are input back into the perceptual system, implementing a sensorimotor negative feed-back control loop that improves recognition performance. It is also suggested that this loop can take place at the segmental level. In the present experiment the units synthesized by the $\beta$-retrieval operation are used to form a negative feed-back loop to the effect that phonetic learning depends not only of the acoustic external input, but is modulated by the predictions made by the system itself. Such predictions can be used as well for phonetic recognition. We leave this question for further research too.

## Experimental setting

The experiments were programmed in Python 3.8 on the Anaconda distribution. The neural networks were implemented with TensorFlow 2.3.0, and most of the graphs produced using Matplotlib. The experiments were run on an Alienware Aurora R5 with an Intel Core i7-6700 Processor, 16 GBytes of RAM and an NVIDIA GeForce GTX 1080 graphics card; and on a 150 Xeon Gold computer, using 22 cores and one NVIDIA Tesla P100 graphic card.

## Data Availability

The datasets used and analysed during the current study are available for academic purposes at http://turing.iimas.unam.mx/~luis/DIME/CORPUS-DIMEX.html and http://www.ciempiess.org. The full code and the results of the experiments, including the six scenarios with their corresponding five learning stages, are available at https://github.com/eam-experiments/dimex.
